# Morphological and molecular insights into the diversity of *Leptoconops* biting midges from a heavily infested Mediterranean area

**DOI:** 10.1016/j.crpvbd.2023.100142

**Published:** 2023-09-23

**Authors:** Carlo Polidori, Paolo Gabrieli, Irene Arnoldi, Agata Negri, Laura Soresinetti, Simone Faggiana, Andrea Ferrari, Federico Ronchetti, Matteo Brilli, Claudio Bandi, Sara Epis

**Affiliations:** a Department of Environmental Science and Policy (ESP), University of Milan, Milan, 20133, Italy; b Department of Biosciences and Pediatric Clinical Research Center “Romeo and Enrica Invernizzi”, University of Milan, Milan, 20133, Italy; c Italian Malaria Network, Inter University Center for Malaria Research, University of Milan, 20133, Milan, Italy; d Department of Biology and Biotechnology, University of Pavia, 27100, Pavia, Italy; e University School of Advanced Studies Pavia, IUSS, 27100, Pavia, Italy; f Biosistemi Srl, Gallarate, 21013, Varese, Italy

**Keywords:** Ceratopogonidae, *Leptoconops*, Tuscany, Blood-sucking midges, Morphological identification, Molecular identification

## Abstract

The genus *Leptoconops* Skuse (Diptera: Ceratopogonidae) are blood-sucking midges known to pester humans and domestic animals. In certain Mediterranean areas, midges occur in large numbers during summer and limit the use of recreational areas, also raising serious health and social concerns. Despite such impact, the diversity and distribution of *Leptoconops* in Maremma Regional Park (Tuscany Region, Italy), a heavily infested area, is not well known, and neither molecular nor detailed morphological studies exist. We sampled adult midge females in six areas and used high-resolution digital stereomicroscopy and scanning electron microscopy to identify species and investigate the morphology of structures involved in host searching/recognition (antennae and maxillary palps) and host attack (mouthparts). We also performed energy-dispersive X-ray spectroscopy to characterize the elemental composition of mouthparts. Finally, the cytochrome *c* oxidase subunit 1 (*cox*1) gene was amplified and sequenced, to confirm species identification of collected specimens. We identified two species: *Leptoconops* (*L*.) *irritans* Noé and *Leptoconops* (*L.*) *noei* Clastrier & Coluzzi, with the former being more frequently sampled than the latter and closer to sea coast and rivers. The antennal segments appeared slightly more globular in *L. noei* than in *L. irritans*. Five types of trichoid, basiconic and chaetic sensilla were found on the antennae, with some differences between the two species. Mouthparts had the labellum visibly larger in *L. noei* compared with *L. irritans*. The maxillary palps possessed a pit filled with bulb-shaped sensilla, which appeared denser in *L. noei* than in *L. irritans*. Mouthpart cuticle included Calcium (Ca) and Aluminum (Al) at small but significant concentrations (0.3–1.0%) in both species. Our results suggest that the limited but appreciable differences in sensory system between the studied species of *Leptoconops* and other Ceratopogonidae may reflect different host or habitat preferences, a scenario potentially suggested also by preliminarily data on their distribution in the studied area. The presence of Ca and Al in the cuticle of mouthparts may help host skin drilling during bite activity. Finally, the gene sequences obtained in this study provide a first reference for future investigations on the taxonomy and dispersal patterns of *Leptoconops* spp. in the Mediterranean area.

## Highlights

• Six sites in the Tuscany Region (Italy) were screened for *Leptoconops* biting midges.
• *Leptoconops noei* and *L. irritans* were identified morphologically and molecularly.
• Sensory organs and mouthparts were studied by high-resolution digital stereomicroscopy.
• Calcium (Ca) and Aluminium (Al) were detected in the mouthparts by energy-dispersive X-ray spectroscopy.

## Graphical abstract

**Figure:**
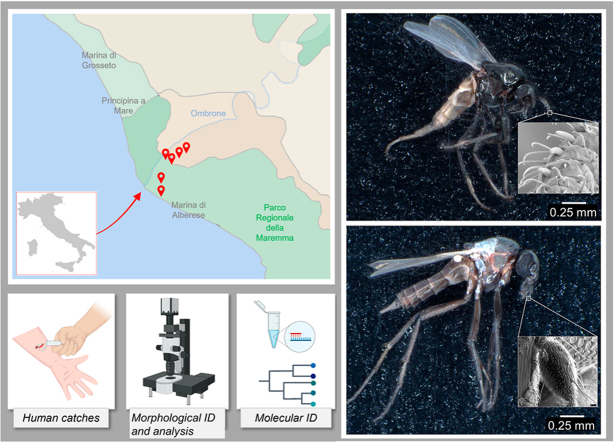

## 1 Introduction

The family Ceratopogonidae (Diptera: Culicomorpha), or biting midges, presents a worldwide distribution and encompasses over 6276 extant species, but most genera within this taxon are still poorly studied (Borkent et al., 2022). At the adult stage, most ceratopogonids feed by sucking biological fluids from other insects, but several species act also as blood-feeders on vertebrates, and a few are competent vectors that transmit pathogens that can cause diseases to domestic animals and humans (Ronderos et al., 2003).

Three subfamilies are recognized in Ceratopogonidae (Ceratopogoninae, Forcipomyiinae and Leptoconopinae), with Leptoconopinae, including the genus *Leptoconops* Skuse, being an ancient lineage mostly distributed in intertropical or subtropical regions. The oldest known fossils date back to 120 million years ago (Borkent, 2001), suggesting that the evolutionary radiation of the group could have started in the Lower Cretaceous or Late Jurassic (Choufani et al., 2011).

The adult gravid females lay eggs in sandy or clay soils (Majori et al., 1971; Raspi et al., 2007), which they break using their very elongated cerci; in such substrates, larvae develop by feeding on decomposing organic matter and microorganisms. Adults then emerge from the soil through necessary soil cracking. The females feed on the blood of vertebrates, including humans, and take a large amount of blood imbibing more than their own body weight (Foulk, 1967; Linley, 1968). Feeding activity is diurnal with maximum biting peak apparently variable among species (Kettle and Linley, 1967; Carrieri et al., 2011).

Eleven species of *Leptoconops* from two subgenera (*Leptoconops* and *Holoconops*) have been reported in Europe (Szadziewski and Borkent, 2004). Of these, five species are present in Italy: *Leptoconops* (*L.*) *irritans* (Noè), *L*. (*L*.) *bezzii* (Noè), *L*. (*L.*) *noei* Clastrier & Coluzzi, *L*. (*L.*) *bidentatus* Gutsevich, and *L*. (*H.*) *kerteszi* Kieffer (Boorman, 1995). These species occur mainly in restricted areas of Tuscany (Central Italy), Basilicata, Puglia (Southern Italy) and Sardinia Island (Coluzzi and Finizio, 1966; Bettini et al., 1969; Majori et al., 1970, 1971; Clastrier and Coluzzi, 1973; De Marzo and Moleas, 1979; Cocchi et al., 1986; Lavagnino et al., 1990; Carrieri et al., 2005; Fausto et al., 2007). As far as we know, none of these species act as vectors of pathogens for humans and other mammals, but females typically fly in large swarms during late spring and summer and impose painful bites (Kettle, 1977). Lesions subsequent to bites are even more severe, especially in children, with strong allergic responses and infections produced by the scratching of long-term itchy papules (Whitsel and Schoeppner, 1966).

In contrast to the recognized economic, social, and health problems associated with the infestation of *Leptoconops* spp. in many areas of the world, there is still a limited knowledge of the life history, behavioral ecology, sensory morphology, and physiology of these insects (Aussel, 1991, 1993a, b; Carrieri et al., 2007, 2011; Raspi et al., 2007; González et al., 2013), particularly compared to *Culicoides* Latreille, the other ceratopogonid genus relevant as a biting midge around the globe (e.g. Sutcliffe and Deepan, 1988; Blackwell et al., 1992; Kline et al., 1994; Rasmussen et al., 2012; Isberg et al., 2016; Mullen and Murphree, 2019).

Here, we present novel information on two species of *Leptoconops* heavily infesting the coastal area of the Maremma Regional Park (Grosseto Province, Central Italy). After a sampling campaign, we determined which species occur in the study area, by both morphological observation and molecular analysis, and then described and discussed certain morphological aspects related to host location (antennae, palpi) and feeding (mouthparts), through stereomicroscopy and scanning electron microscopy. To date, no studies have been carried out for any Italian species of *Leptoconops* at this level of morphological and genetic detail. We argue that our investigation may revamp the interest in studying the biology of these midges, which could contribute to the development of methods and strategies for the control of these pests.

## 2 Materials and methods

### 2.1 Field collection and morphological identification of midges

Females of *Leptoconops* were collected in the Maremma Regional Park at six sampling points (Fig. 1A): Agriturismo Sagrado (site A) (1 km from the small town Alberese, 42°41′13.3″N, 11°06′45.0″E), Marina di Alberese (site B) (on the coast, 42°38′58.74″N, 11°2′10.392″E), one site close to the River Ombrone (site C) (42°41′41.883″N, 11°4′52.705″E), and three sites along the road that connect Alberese to the sea coast: site D (42°40′57.4″N, 11°05′16.6″E), site E (42°40′49.0″N, 11°04′28.1″E) and site F (42°40′28.827″N, 11°3′23.839″E). About 1–4 km separated the sampling sites (Fig. 1A). Maremma Regional Park is rich in clayey-silty soils, which are optimal for larval development. The Park is also rich in both wildlife and livestock and horses reared at local farms, which is an optimal situation for adult midge feeding. Hence, this area is well known to present heavy infestations of *Leptoconops* spp. during the summer season (Majori and Bettini, 1971).

**Fig. 1:**
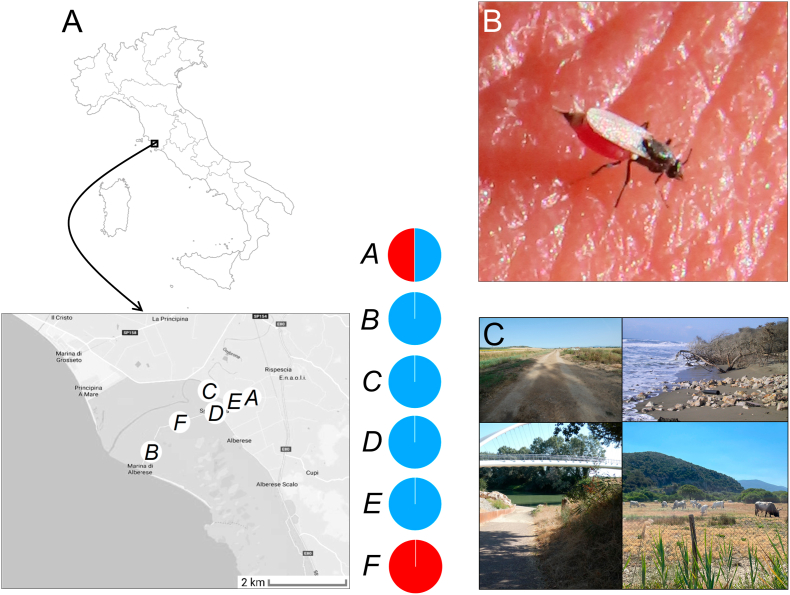
Study sites and relative abundances of *Leptoconops* species. **A** Location of the study area and the six sampling sites (*A-F*). On the right of the latter map, graphs depicting the relative abundance of the two collected species (*Leptoconops irritans* (*blue*) and *Leptoconops noei* (*red*) are shown. **B***Leptoconops irritans* female while feeding on a human hand during the sampling. **C** Examples of habitat types at the sampling sites (from top left to bottom right in clockwise direction: *A, B, C, F*).

Females were collected by placing Eppendorf tubes on midges during their biting/blood-sucking activity on some volunteers involved in this study (Fig. 1B), a technique revealed to be adequate to sample large numbers of *Leptoconops* individuals in a relatively short time (Perich et al., 1995; Carrieri et al., 2007; González et al., 2013). Sampling took place from 10.00 h to 17.00 h, from 23 June to July 10, 2021 (sites A, B and C), from 7 to June 12, 2022 (sites A, B, C, D, E and F), and from 10 to July 13, 2022 (sites A, B, C, D, E and F). Upon collection, specimens were frozen at −20 °C and then submerged in 99% ethanol. Each Eppendorf tube was labeled with a unique code reporting the sampling point from where the specimens came.

The collected individuals were first observed using a stereomicroscope in order to identify them at the species level (Leica M50, Leica, Wetzlar, Germany) by using the taxonomic keys of Clastrier and Coluzzi (1973). Secondly, for a selected number of specimens, we used the high-precision digital stereomicroscope VHX-7000 (Keyence, Osaka, Japan), able to overcome the limitations of traditional optical microscopes by providing high-resolution imaging, wide depth of field, and integrated 2D/3D measurements. Thirdly, a selected number of specimens were observed and pictured using a scanning electron microscope (SEM) (Zeiss LEO 1430, Zeiss, Oberkochen, Germany). For the SEM analysis, specimens were sputter-coated with gold, mounted on aluminum stubs and finally placed in the SEM for visualization, following our previous similar studies on small dipterans (Polidori and Wurdack, 2019). High vacuum conditions (resolution: 3.0 nm at 30 kV (Secondary electrons-SE), 10 nm at 3 kV (SE), and 4.0 nm at 30 kV (Backscattered electrons-BSEs) were used. The accelerating voltage was 26 kV, the high vacuum was 53.3–66.6 Pa, and the working distance was 10 mm.

### 2.2 DNA extraction, molecular identification and phylogenetic analyses

Each specimen was washed in phosphate-buffered saline and cut by a scalpel in an extraction solution (15 μl, Tris-HCl 10 mM, pH 8.4). Then, samples were first incubated at 90 °C for 10 min and subsequently at 45 °C for 3 h with proteinase K (50 units/ml, New England Biolabs, Ipswich, Massachusetts), followed by a final inactivation of the enzyme at 90 °C for 5 min. After centrifugation, 2 μl of each sample was used for amplification of the mitochondrial gene cytochrome *c* oxidase subunit 1 (*cox*1) through polymerase chain reaction (PCR) using the primers LCO1490 and HCO2198 (Folmer et al., 1994). PCR products were visualized on agarose gel, purified by gel extraction, and sequenced (Eurofins Genomics, Ebersberg, Germany). Sequences were deposited in the GenBank database under the following accession numbers (included in the phylogenetic tree): OM672379-OM672398 (Table 1).

**Table 1:** GenBank accession numbers of the *cox*1 sequences.

Species	Accession number
*Leptoconops noei*	OM672379-OM672383
*Leptoconops irritans*	OM672384-OM672399

The newly generated sequences and *cox*1 sequences of closely related species (Ceratopogonidae) were retrieved from the GenBank database by using the nucleotide Basic Local Alignment Search Tool (nBLAST: https://blast.ncbi.nlm.nih.gov/Blast.cgi) and aligned using MUSCLE, with default parameters for nucleotide sequences. After manual editing, IQ-Tree software was used to infer the best phylogenetic tree for the data by maximum likelihood; bootstrap support values were determined by 1000 replicates. The best-fit model of nucleotide substitution was GTR + I + F + G4 (Tavaré, 1986).

### 2.3 Energy-dispersive X-ray spectroscopy (EDS) analysis

A JEOL JSM-IT500 LV Scanning Electron Microscope (SEM) (JEOL Ltd., Tokyo, Japan), equipped with Backscattered (BSE) and Secondary Electrons (SE) detectors, and coupled with energy dispersive X-ray spectrometry (EDS), was employed to perform semi-quantitative chemical micro-analysis through EDS, at the Department of Earth Sciences “Arditio Desio” of the University of Milano (ESD-UniMI). The operating conditions were: vacuum mode, 20 kV accelerating voltage and 10 mm working distance. ZAF correction was applied to the chemical data, as implemented in the JEOL suite of programs. We performed such analysis because, in previous studies, the cuticle of insect structures involved in penetrating a substrate (e.g. ovipositors) are often enriched in transition, post-transition and/or alkaline-earth metals such as Zinc (Zn), Manganese (Mn), Aluminum (Al), Calcium (Ca) and Magnesium (Mg) (Polidori and Wurdack, 2019; Lehnert et al., 2019; Polidori et al., 2020). These metals were proved, through a combination of EDS and nanoindentation, to effectively increase cuticle hardness in these structures (Cribb et al., 2008; Li et al., 2020). Hence, the occurrence of metals in *Leptoconops* spp. mouthparts may suggest an adaptation to host skin-drilling.

For each specimen, we performed a point analysis, in which metal concentration was obtained through X-rays directed to a single point on the labrum, maxilla, mandible and hypopharynx of three individuals *per* species. The weight percentage of the sigma value (i.e. the error in the weight percent concentration at the 1 sigma level) was used to determine whether the element is below the detection limits of the sample analysis (Duncumb, 1994). In particular, an element was considered as significantly occurring in the cuticle if the observed weight percentage of the metal was greater than three times the weight percentage of the sigma value (Polidori et al., 2013, 2020).

## 3 Results

### 3.1 Species identification based on morphology

All 241 sampled adults resulted to be *Leptoconops* spp. females after a first inspection under the stereomicroscope. These individuals were then mounted, and further morphologically examined.

According to the description and keys of Clastrier and Coluzzi (1973), the collected samples belong to the subgenus *Leptoconops* (*Leptoconops*) since they possess 14 antennal segments and not 13 segments (i.e. 12 and not 11 flagellomers, *plus* scape and pedicel) as in the other subgenus recorded in Italy, *Leptoconops* (*Holoconops*). Furthermore, members of the subgenus *Leptoconops* (*sensu stricto*) have maxillary palpi with several sensory pits (palpal pits (PP), see below) (De Meillon and Wirth, 1991), while members of the subgenus *Holoconops* have palpi with a deep enclosed sensory pit (Raspi et al., 2007). A first examination using the keys of Clastrier and Coluzzi (1973) led initially to three possible species of *Leptoconops* (*s.s.*): *Leptoconops irritans*, *L. noei* and *L. bezzii*.

**Fig. 2:**
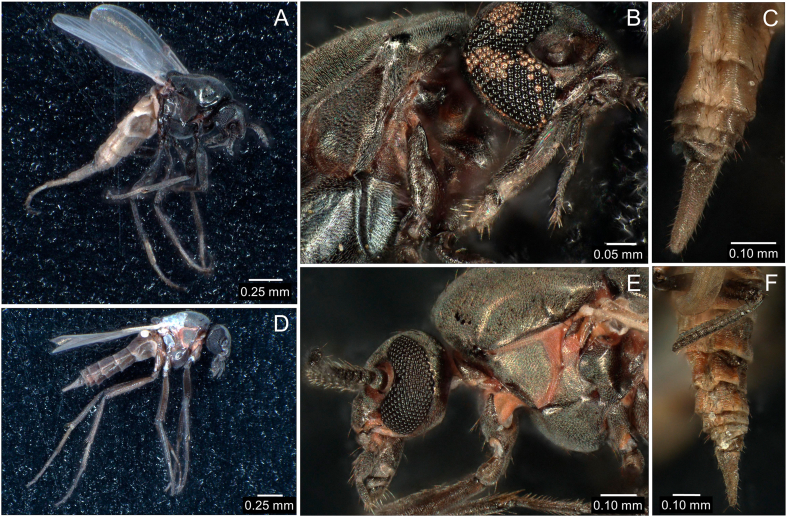
Microphotographs of *Leptoconops* spp. taken under a high-resolution stereomicroscope. **A**-**C***Leptoconops irritans*. **D**-**F***Leptoconops noei.* Whole body (**A**, **D**), head and thorax (**B**, **E**), posterior abdominal segments (**C**, **F**) of the females of the two species are shown.

*Leptoconops irritans* and *L. noei* were recognized as the only two species occurring in the sample, due to the following observations. Unlike *L.* *bezzii*, *L. irritans* females have: antennae covered by denser, more rigid and spinier setae (chaetic sensilla (Cs), see below); maxillary palps longer than the proboscis; and longer and slender proboscis (Fig. 2A–E)*.* Compared with *L.* *bezzii*, *L. noei* have the ventral side of the antennae with a pair of longer, thinner and sharper blunt trichoid sensilla (StB, see below), curved towards the antennal longitudinal axis (in a way that they roughly resemble S-shaped cow horns). The two StB are diametrically opposite on the distal antennal segments but tend to get closer to each other on the proximal segments. The ventral side of the antennae, furthermore possesses, on all segments, denser and thicker sharp trichoid sensilla (StS, see below) compared with *L. bezzii*. Compared with *L.* *bezzii*, *L. noei* has palps longer than the proboscis, passing it for half of the last segment of the palp. In frontal view, the third palp article has two StS aligned on the inner lateral portion, one StS placed within the large sensory pit of the palp and three StS aligned on the external lateral portion. The palpal pit has fewer bulb-shaped sensilla (Bss, see below) compared with *L. bezzii*. The labium of *L. noeii*, differently from *L. bezzii*, covers longitudinally the labrum, the maxillae and the mandibles.

*Leptoconops irritans* and *L. noei* could be distinguished essentially because *L. noei* has the palpal segments 4 and 5 fused, appearing four-segmented (Fig. 2B, E). *Leptoconops noei* seems also slightly larger overall compared with *L. irritans* (wing length: 1.0–1.3 *vs* 0.9–1.0 mm) (Fig. 2A, Supplementary Fig. S1) but has a slightly shorter ovipositor (relative to wing length) compared with *L. irritans* (ovipositor/wing length ratio: 0.15 *vs* 0.20) (Fig. 2C, F). Further inspection of the SEM pictures revealed additional characteristics of antennae and mouthparts never appreciated and considered before, that could be useful in discriminating these two species (see below).

### 3.2 Morphological remarks for *L*. *irritans* and *L*. *noei*

**Fig. 3:**
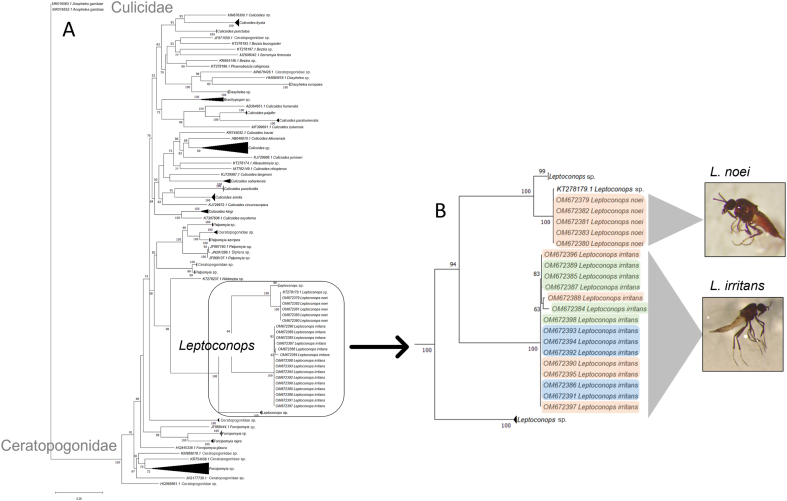
Phylogenetic trees for *cox*1 sequence data. Trees were built using IQ-Tree software to infer the best phylogenetic tree for the data by maximum likelihood; bootstrap support values were determined by 1000 replicates. The best-fit nucleotide substitution model estimated was GTR + I + F + G4. **A** Entire tree. **B** A magnified image of the part of the tree including *Leptoconops*. Different colors refer to different sampling sites.

**Fig. 4:**
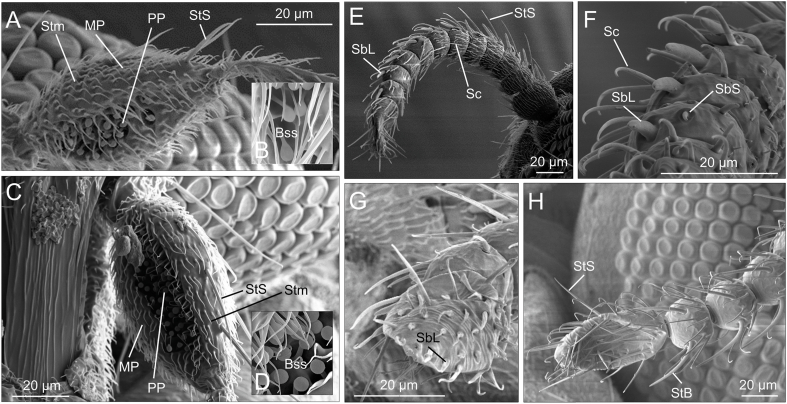
SEM micrographs of the sensory system of *Leptoconops irritans* (**A**-**B**, **E**, **F**) and *Leptoconops noei* (**C**-**D**, **G**, **H**). **A**-**D** Palpi. **E**-**H** Antennae. *Abbreviations*: MP, maxillary palp; PP, palpal pit; Bss, bulb-shaped sensilla; Stm, microtrichoid sensilla; StS, sharp trichoid sensilla; StB, blunt trichoid sensilla; SbL, long basiconic sensilla; SbS, short basiconic sensilla; Sc, chaetic sensilla.

**Fig. 5:**
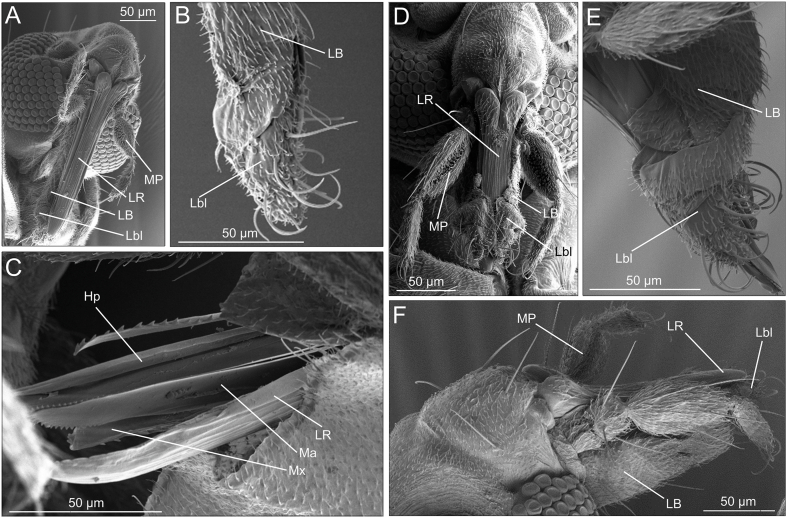
SEM micrographs of the mouthparts of *Leptoconops irritans* (**A**–**C**) and *Leptoconops noei* (**D**–**F**). *Abbreviations*: MP, maxillary palp; LB, labium; Lbl, labellum; LR, labrum; Mx, maxilla; Ma, mandible; Hp, hypopharinx.

The most distal (14th) antennal segment (i.e. the 12th flagellomer) was similar in shape in the two species (Fig. 2, Fig. 3). However, the remaining flagellomers appeared slightly more globular in *L. noei* than in *L. irritans* (Fig. 4). Five types of sensilla were found on the antennae of both species: sharp trichoid sensilla (StS); blunt trichoid sensilla (StB); long basiconic sensilla (SbL); short basiconic sensilla (SbS); and chaetic sensilla (Sc) (Fig. 4). SbL were found only on the distal flagellomer in *L. noei*, while one SbL was found on each segment from 13th to 2nd in *L. irritans.* On the other hand, two long StB were found from 13th to 2nd segment only in *L. noei* (Fig. 4). Mouthparts are composed of labium (LB), labellum (Lbl), labrum (LR), maxilla (Mx), mandible (Ma) and hypopharynx (Hp) (Fig. 5). Labellum seemed to differ between the two species, being visibly larger (and covering a greater part of the LR) in *L. noei* (Fig. 5). The maxillary palps (MP) possess a palpal pit (PP) filled with bulb-shaped sensilla (Bss), denser in *L. noei* than in *L. irritans* (Fig. 5), and they were covered in both species by small microtrichoid sensilla (Stm) and sharp trichoid sensilla (StS) (Fig. 5).

**Fig. 6:**
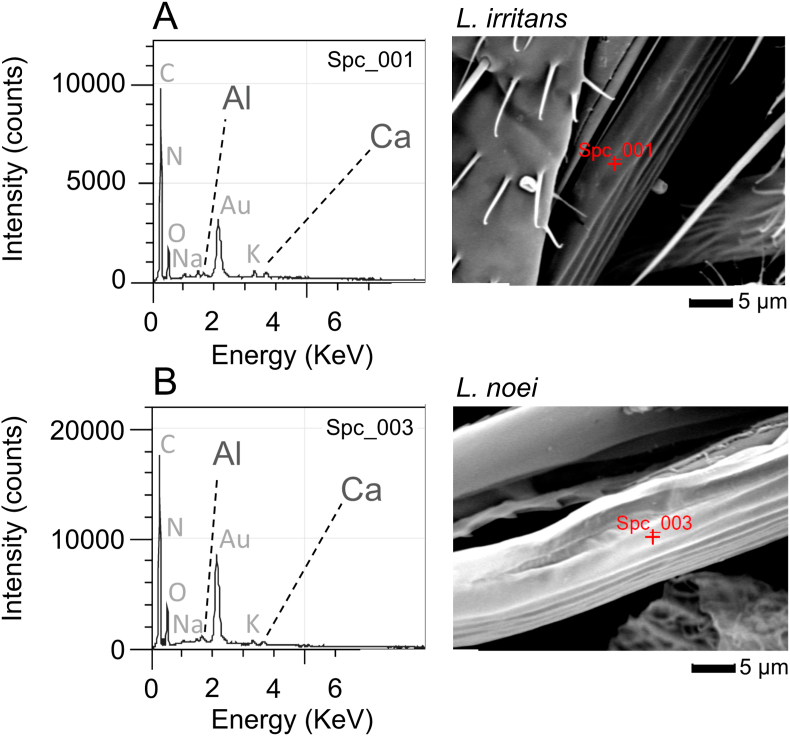
Representative spectra from X-ray energy dispersive (EDS) analysis of the mouthparts of *Leptoconops irritans* (**A**) and *Leptoconops noei* (**B**). Spectra were generated with a primary electron beam energy of 20 keV. The peaks for the detected metals (Ca and Al) are indicated; other elements were also detected (indicated in grey).

The EDS analysis showed that the mouthpart cuticle included Calcium (Ca) and Aluminum (Al) at small but detectable concentrations (0.3–1.0%) in both species (Fig. 6).

### 3.3 Distribution of the two species in the area of study

*Leptoconops irritans* was by far the most abundant species (*n* = 218 individuals) and was the only species collected at four out of the six sampling sites (Fig. 1A). The only two sites where *L. noei* was collected (sites A and F) were located at more than 3 km from the seacoast and the river coast. At both sites A and F, numerous cattle farms are present (with the animals either kept in cattle sheds or in open pasture spaces) (Fig. 1C). On the other hand, the largest number of *L. irritans* individuals (*n* = 173) were sampled close to sea coast and river coasts (sites B and C) (Fig. 1A, C), where cattle farms are not present. Moreover, the coastal sites were characterized by highly sandy soils, while the more inner sites had a harder soil.

### 3.4 *Leptoconops* species assignation through DNA sequence analysis

A subset of the morphologically identified specimens of *Leptoconops* was molecularly analyzed using the barcode region of the *cox*1 gene. Twenty sequences were generated and deposited in GenBank (Table 1): 5 for *L. noei* (OM672379-OM672383) and 15 for *L. irritans* (OM672384-OM672398). Until the submission of the sequences derived from our samples, the GenBank database lacked *cox*1 sequence data for *L. irritans* and *L. noei*.

The newly generated *L. noei* sequences were identical and displayed the highest similarity to a *cox*1 sequence of *Leptoconops* sp. available in the GenBank database (99.84%, KT278179.1). The sequences of the samples morphologically identified as *L. irritans* showed higher variability and were similar to two *cox*1 sequences of *Leptoconops* sp. in the GenBank database (from 83.12 to 82.79%, KT278284.1 and KT278283.1). The phylogenetic tree shown in Fig. 3, clearly shows that our sequences cluster with other sequences of *Leptoconops* spp. with 100% bootstrap support. Sequences of *L.* *irritans* formed a distinct cluster (100% bootstrap), while sequences of *L. noei* clustered with other sequences classified as *Leptoconops* sp. (GenBank: KT278173.1, KT278285.1 and KT278179.1).

## 4 Discussion

In Italy, information on species composition and abundance of midges of the genus *Leptoconops* is limited. Few publications provide an “inventory” of *Leptoconops* species, whose presence has been recorded in several regions (Coluzzi and Finizio, 1966; Bettini et al., 1969; Majori et al., 1970, 1971; Clastrier and Coluzzi, 1973; De Marzo and Moleas, 1979; Cocchi et al., 1986; Lavagnino et al., 1990; Carrieri et al., 2005; Fausto et al., 2007). In this study, we investigated species presence and relative abundance of these midges in the highly touristic area of Maremma Regional Park, one of the most infested areas in Italy.

The coastal area of the province of Grosseto (Maremma, Italy) has favorable environmental conditions for the development of ceratopogonids of the genus *Leptoconops*. Indeed, these territories are densely populated by cattle, horses, and wildlife that can favor the development of different species of ceratopogonids of the genera *Leptoconops* and *Culicoides*. As mentioned above, the occurrence in large numbers during the activity peak of the year makes these minute midges extremely annoying, limiting the use of some areas as tourist attractions and recreational locations, and producing skin irritation or even severe dermatitis (Majori and Bettini, 1971; Strickman et al., 1995). However, no effective control strategy has been achieved yet and applied studies are far from pointing to practical solutions to contain these pests (Aussel, 1991, 1993a, b; Belardinelli et al., 2010). Proposed methods to control *Leptoconops* include an artificial alteration of the soil moisture to prevent cracking, but it seems not always easy to achieve (Whitsel et al., 1961; Foulk, 1966).

Accurate species identification is the first step needed for the study of *Leptoconops* species. In particular, the combination of morphological and molecular methods makes their identification easier and will likely facilitate future studies by researchers who are not taxonomists. Here, we performed a morphological study on two species of *Leptoconops* midges (i.e. *L*. *irritans* and *L*. *noei*) to a deeper detail than in previous studies, focusing on structures that are likely important in their behavioral ecology (sensory organs and mouthparts). We emphasize that organs and structures involved in the behavioral ecology of congeneric species are likely to present interspecific variation. By focusing on these structures, it is possible to uncover new morphological characters, useful both in the identification of the species and in highlighting eco-ethological differences among species. Moreover, for the first time, the two species have also been characterized at the molecular level, obtaining specific *cox*1 sequences that contributed to the discrimination of these species, revealing their positioning into two separate clades. It would be interesting to obtain other *Leptoconops* sequences from different species and populations collected in Italy to investigate their relationships.

Considering morphology, we described a few characteristics previously overlooked in studies on the genus *Leptoconops*, regarding structures involved in host detection/recognition and feeding. First, we observed new details on the antennal and palpal sensory structures, which both present differences between the two examined species. While the gross morphology of the antennae is very similar in the two species (with only a slightly more globular shape of most segments in *L. noei* and *L. irritans*), overall also resembling those of the phylogenetically closely related midge genus *Culicoides* Latreille (Blackwell et al., 1992; Felippe-Bauer et al., 1989; Messaddeq et al., 1989, 1990), certain sensillar types differ in shape and/or distribution, as also observed among species of *Culicoides* (Kline and Axtell, 1999). For example, SbL (long basiconic sensilla) were found only on the distal segment in *L. noei*, while one SbL was found on each segment from 13th to 2nd in *L. irritans*. In *Culicoides*, the mean number of antennal sensilla basiconica does not appear to be associated with host preference (Braverman and Hulley, 1979), despite their number being rather different in different species (Kline and Axtell, 1999). Both *L*. *irritans* and *L. noei* attack humans, but perhaps the latter species also largely uses other mammals, such as bovines, which are abundant at the collection sites. On the other hand, *L. irritans* seems to be more abundant in areas with a minor density of livestock and can possibly be associated with humans in highly touristic sites such as beaches.

Probably because of these potential niche differences, long StB (blunt trichoid sensilla) were found on the 13th to 2nd segment only in *L. noei*. In the genus *Culicoides*, StB occurs in all investigated species along all antennal flagellomers, thus making *L. noei* more similar to *Culicoides* spp. than to *L. irritans* in this aspect. In some blood-feeding Diptera (including mosquitoes and *Culicoides*), StB seem to be the principal olfactory sensory organs, that, at least in some species, mediate responses to repellents (Steward and Atwood, 1963) and/or provide an odor-trapping net for long-range host-derived odors (Blackwell et al., 1992). Another sensillar type, the StS (sharp trichoid sensilla) did not seem to differ in number or distribution between the two investigated *Leptoconops* species, while being associated with host location/detection in mosquitoes and *Culicoides* spp. (Steward and Atwood, 1963; Blackwell et al., 1992). Further studies are necessary to understand if such differences in SbL and StB between the two species may reflect possible host preferences.

Concerning the maxillary palps, *Leptoconops* spp. present a palpal pit (PP) filled with bulb-shaped sensilla (Bss), apparently slightly denser in *L. noei* than in *L. irritans*. These structures present slight differences in *Culicoides* spp., and the mean number of bulb-shaped sensilla on the palpal pits, which appear to have an olfactory function (Rowley and Cornford, 1972), seems to be related to host preference (Braverman and Hulley, 1979). The fact that *L. noei* possesses not only more StB, but also more Bss, further suggests some degree of differences in host range preference between the two studied species. Future behavioral and physiological data will be necessary to test this hypothesis. In both species, maxillary palps were also found to be covered by small microtrichoid sensilla (Stm) and sharp trichoid sensilla (StS), similar to what was observed in *Culicoides* spp. (Kline and Axtell, 1999).

We also detected, for the first time in Ceratopogonidae, the presence of two metals, Al and Ca, in the cuticle of mouthparts. Ca is known to occur in the cuticle of some insects, including species of Diptera (Quicke et al., 2004; Garcia-Guinea et al., 2011; Polidori and Wurdack, 2019; Li et al., 2020). The function of Ca in insect cuticle is not well known, but recent studies show that it can increase cuticle hardness (Li et al., 2020), as it does in crustaceans (Roer and Dillaman, 1984) and other non-insect arthropods (e.g. Thorez et al., 1992). On the other hand, Al was rarely reported in insects. For example, it was recently detected in the ovipositor of cicadas (Lehnert et al., 2019). The rarity of Al detection in insects, however, could derive from the fact that studies are generally focused on other metals, such as Zn and Mn (and hence did not quantify Al), since they are more clearly related to cuticle hardness than on the actual absence of this metal in the cuticle. The presence of this metal might be also related to the life-cycle of the species, which feeds in the soil during the larval stage and might accumulate rare metals from this substrate. Further studies are necessary to evaluate how the abundance of these metals may improve the use of mouthparts during biting and blood-feeding in *Leptoconops*.

Studying *Leptoconops* insects in touristic areas is advisable due to the impact on human well-being. In fact, large swarms, formed by hundreds or even thousands of insects, feed on the blood of mammals, including humans, causing allergic reactions, especially in children. Furthermore, *Leptoconops* spp. might play a role as vectors for various pathogens, such as viruses or protozoans, an issue that is discussed in several papers, but rarely investigated. Other ceratopogonids, such as *Culicoides* midges, are able to transmit viruses (bluetongue and Schmallenberg viruses) and protozoans, in particular of the family Trypanosomatidae. Like in *Culicoides*, the females of the genus *Leptoconops* feed on reptiles, birds, and mammals in general (Mullens et al., 1997; Mullen, 2002; Szadziewski and Poinar, 2005), but sound evidence for the involvement of these insects in the transmission of pathogens to vertebrates is still lacking.

Despite the lack of evidence for the involvement of *Leptoconops* in the transmission of pathogenic microorganisms to humans or other vertebrates, it is interesting that a classical study by the amber specialist George Poinar revealed the presence of *Trypanosoma*-like protists in the gut, salivary glands, and salivary secretions of *Leptoconops nosopheris* Poinar, a fossil midge in Early Cretaceous amber (Poinar, 2008). The presence of trypanosomatid microorganisms in both the gut and salivary glands of this ancient midge is highly suggestive of the role of the midge itself in the transmission of these microorganisms. Aside from these paleontological insights for a role in trypanosomatid protozoan transmission, *Leptoconops* midges could possibly be involved also in the transmission of filarial nematodes. This is suggested by a study on *Leptoconops bequaerti* (Kieffer), as a potential further vector of the *Mansonella ozzardi* Manson (Lowrie et al., 1983), in addition to the typical vectors of the genera *Culicoides* and *Simulium.*

## 5 Conclusions

Our study revealed for the first time limited but appreciable differences in some components of the peripheral sensory system between the studied species and between *Leptoconops* and other Ceratopogonidae. These differences merit further investigations to test if they reflect different host or habitat preferences, a scenario preliminarily suggested also by a certain degree of ecological niche segregation, as emerged from our field data. On the other hand, the presence of Ca and Al in the cuticle of mouthparts seems to follow a similar pattern in the two studied species, likely because the host skin needs similar adaptations to be efficiently drilled during bite activity. Finally, our molecular analysis is a first step toward the study of the dispersal patterns of these species, in the Mediterranean area. Future studies should also address the issue of whether *Leptoconops* spp. act as vectors of pathogens for domestic animals and humans.

## Funding

This work was partially supported by EU funding within the NextGeneration EU-MUR PNRR Extended Partnership initiative on Emerging Infectious Diseases (Project no. PE00000007, INF-ACT) and by the EU funding within the NextGeneration EU-MUR PNRR, Investment 1.5 “Innovation Ecosystems” (Project MUSA).

## Ethical approval

Not applicable.

## CRediT authorship contribution statement

**Carlo Polidori:** Conceptualization, Investigation, Visualization, Resources, Writing – original draft, Writing – review & editing. **Paolo Gabrieli:** Investigation, Visualization, Writing – review & editing. **Irene Arnoldi:** Investigation, Visualization, Writing – review & editing. **Agata Negri:** Investigation, Writing – review & editing. **Laura Soresinetti:** Investigation, Writing – review & editing. **Simone Faggiana:** Visualization, Writing – review & editing. **Andrea Ferrari:** Investigation, Writing – review & editing. **Federico Ronchetti:** Investigation, Writing – review & editing. **Matteo Brilli:** Methodology, Writing – review & editing. **Claudio Bandi:** Writing – review & editing. **Sara Epis:** Conceptualization, Investigation, Visualization, Resources, Writing – original draft, Writing – review & editing.

## Declaration of competing interests

The authors declare that they have no known competing financial interests or personal relationships that could have appeared to influence the work reported in this paper.

## Figures and Tables

**figs1:**
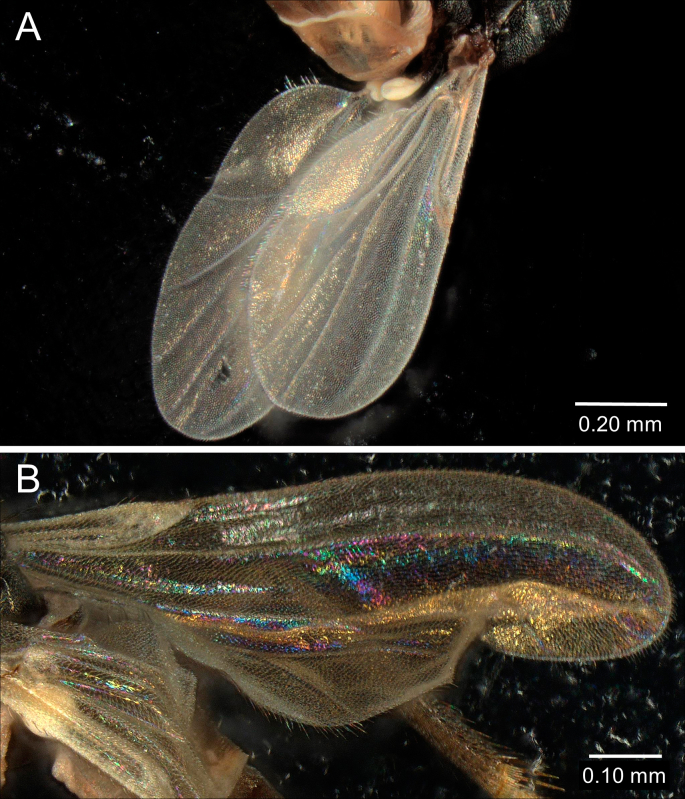
Wings of *Leptoconops irritans* (A) and *L. noei* (B). The wings of *Leptoconops* spp. were microphotographed under a high-precision digital stereomicroscope VHX-7000 (Keyence, Japan).

## Data Availability

The dataset supporting the conclusions of this article is included within the article. Nucleotide sequences generated in this study were deposited in the GenBank database under the accession numbers OM672379-OM672398.

## References

[bib1] Aussel J.P. (1991).

[bib2] Aussel J.P. (1993). Ecology of the biting midge *Leptoconops albiventris* in French Polynesia. II. Location of breeding sites and larval microdistribution. Med. Vet. Entomol..

[bib3] Aussel J.P. (1993). Ecology of the biting midge *Leptoconops albiventris* in French Polynesia. III. Influence of abiotic factors on breeding sites. Towards ecological control?. Med. Vet. Entomol..

[bib4] Belardinelli M., Cocchi M., Raffaelli I., Guerra L., Tamburro A., Fausto A.M. (2010). Results preliminary attempts to control the larvae of *Leptoconops* (*Holoconops*) *kerteszi* Kieffer, 1908 (Diptera, Ceratopogonidae) in the coastal wetlands of Tuscany, Italy. Ann. Trop. Med. Parasitol..

[bib5] Bettini S., Majori G., Finizio E., Pierdominici G. (1969). Ricerche sui Ceratopogonidi nel Grossetano. Nota II: Identificazione dei focolai di *Leptoconops bezzii* Noè, 1907. Riv. Parassitol..

[bib6] Blackwell A., Mordue A.J., Mordue W. (1992). Morphology of the antennae of two species of biting midge: *Culicoides impunctatus* (Goetghebuer) and *Culicoides nubeculosus* (Meigen) (Diptera, Ceratopogonidae). J. Morphol..

[bib7] Boorman J., Boormann J., Coluzzi M., Contini C., Ferrarese U., Rivosecchi L., Rossaro B., Sabatini A., Wagner R. (1995). Diptera Culicomorpha. In: Minelli, A., Ruffo, S., La Posta, S (Eds.). Checklist Delle Specie Della Fauna Italiana.

[bib8] Borkent A. (2001). *Leptoconops* (Diptera: Ceratopogonidae): the earliest extant lineage of biting midge, discovered in 120–122 million-year-old Lebanese amber. Am. Mus. Novit..

[bib9] Borkent A., Dominiak P., Díaz F. (2022). An update and errata for the catalog of the biting midges of the world (Diptera: Ceratopogonidae). Zootaxa.

[bib10] Braverman Y., Hulley P.E. (1979). The relationship between the numbers and distribution of some antennal and palpal sense organs and host preference in some *Culicoides* (Diptera: Ceratopogonidae) from Southern Africa. J. Med. Entomol..

[bib11] Carrieri M., Montemurro E., Valentino S., Bellini R. (2007). Study on the flying height of *Leptoconops noei* and *Leptoconops irritans* in southern Italy. Bull. Insectol..

[bib12] Carrieri M., Montemurro E., Valentino S.V., Bellini R. (2011). Influence of environmental and meteorological factors on the biting activity of *Leptoconops noei* and *Leptoconops irritans* (Diptera: Ceratopogonidae) in Italy. J. Am. Mosq. Control Assoc..

[bib13] Carrieri M., Valentino S.V., Montemurro E. (2005). Lotta ecocompatibile agli insetti ematofagi nel Metapontino. Quaderni dell'Ambiente, Provincia di Mater..

[bib14] Choufani J., Azar D., Perrichot V., Soriano C., Tafforeau P., Nel A. (2011). The genus *Leptoconops* Skuse (Diptera: Ceratopogonidae) in early cretaceous charentese amber. Palaeobiodivers. Palaeoenviron..

[bib15] Clastrier J., Coluzzi M. (1973). *Leptoconops* (*Leptoconops*) *bezzii* (Noè, 1905) et *Leptoconops* (*Leptoconops*) *noei* n. sp. (Diptera, Ceratopogonidae). Parassitologia.

[bib16] Cocchi M., Menichetti D., Vichi E., Tamburro A., Gatti L. (1986). Composizione e distribuzione di una popolazione larvale naturale di *Leptoconops* (*Holoconops*) *gallicus* Clastrier, 1973 (Diptera, Ceratopogonidae). Ann. Ist. Super Sanita.

[bib17] Coluzzi M., Finizio E. (1966). Il problema degli insetti ematofagi nella zona litoranea della Provincia di Grosseto. Riv. Malariol..

[bib18] Cribb B.W., Stewart A., Huang H., Truss R., Noller B., Rasch R., Zalucki M.P. (2008). Insect mandibles - comparative mechanical properties and links with metal incorporation. Naturwissenschaften.

[bib19] De Marzo L., Moleas T. (1979). Atti VIII Simposio Nazionale Sulla Conservazione Della Natura, Bari.

[bib20] De Meillon B., Wirth W.W. (1991). The genera and subgenera (excluding *Culicoides*) of the afrotropical biting midges (Diptera: Ceratopogonidae). Ann. Natal. Mus..

[bib21] Duncumb P. (1994). Correction procedures in electron probe microanalysis of bulk samples. Mikrochim. Acta.

[bib22] Fausto A.M., Belardinelli M., Cocchi M., Tamburro A. (2007).

[bib23] Felippe-Bauer M.L., Bauer P.G., Silva Filho F.C. (1989). Scanning electron microscopy of the antennal sensilla in female *Culicoides paraensis* (Diptera: Ceratopogonidae). Mem. Inst. Oswaldo Cruz.

[bib24] Folmer O., Black M., Hoeh W., Lutz R., Vrijenhoek R. (1994). DNA primers for amplification of mitochondrial cytochrome *c* oxidase subunit I from diverse metazoan invertebrates. Mol. Mar. Biol. Biotechnol..

[bib25] Foulk D. (1966). Drainage of a desert spring creek for control of *Leptoconops kerteszi* (Diptera: Ceratopogonidae). Mosq. News.

[bib26] Foulk J.D. (1967). Bloodmeal size of *Leptoconops kerteszi* (Diptera: Ceratopogonidae). Mosq. News.

[bib27] Garcia-Guinea J., Jorge A., Tormo L., Furio M., Crespo-Feo E., Correcher V. (2011). Ossification vesicles with calcium phosphate in the eyes of the insect *Copium teucrii* (Hemiptera: Tingidae). Sci. World J..

[bib28] González M., Lopez S., Goldarazena A. (2013). New record of the biting midge *Leptoconops noei* in Northern Spain: Notes on its seasonal abundance and flying height preference. J. Insect Sci..

[bib29] Isberg E., Bray D.P., Birgersson G., Hillbur Y., Ignell R. (2016). Identification of cattle-derived volatiles that modulate the behavioral response of the biting midge *Culicoides nubeculosus*. J. Chem. Ecol..

[bib30] Kettle D.S. (1977). Biology and bionomics of bloodsucking ceratopogonids. Annu. Rev. Entomol..

[bib31] Kettle D.S., Linley J.R. (1967). The biting habits of *Leptoconops bequaerti*. II. Effect of meteorological conditions on biting activity; 24 hour and seasonal cycles. J. Appl. Ecol..

[bib32] Kline D.L., Axtell R.C. (1999). Sensilla of the antennae and maxillary palps of *Culicoides hollensis* and *C. melleus* (Diptera: Ceratopogonidae). J. Med. Entomol..

[bib33] Kline D.L., Hagan D.V., Wood J.R. (1994). *Culicoides* responses to 1-octen-3-ol and carbon dioxide in salt marshes near Sea Island, Georgia. U.S.A. Med. Vet. Entomol..

[bib34] Lavagnino A., Maroli M., Majori G., Cavallini C., Morello R. (1990). Presenza in Sicilia di *Leptoconops noei* (Diptera, Ceratopogonidae). Parassitologia.

[bib35] Lehnert M.S., Reiter K.E., Smith G.A., Kritsky G. (2019). An augmented wood-penetrating structure: *Cicada* ovipositors enhanced with metals and other inorganic elements. Sci. Rep..

[bib36] Li H., Sun C.Y., Fang Y., Carlson C.M., Xu H., Ješovnik A. (2020). Biomineral armor in leaf-cutter ants. Nat. Commun..

[bib37] Linley J.R. (1968). Autogeny and polymorphism for wing length in *Leptoconops becquaerti* (Kieff.) (Diptera: Ceratopogonidae). J. Med. Entomol..

[bib38] Lowrie R.C., Raccurt C.P., Eberhard M.L., Katz S.P. (1983). Assessment of *Leptoconops bequaerti* as a potential vector of *Mansonella ozzardi* in Haiti. Am. J. Trop. Med. Hyg..

[bib39] Majori G., Bernardini F., Bettini S., Finizio E., Pierdominici G. (1971). Ricerche sui Ceratopogonidi nel Grossetano: nota V: caratteristiche dei focolai di *Leptoconops* spp. Riv. Parassitol..

[bib40] Majori G., Bettini S. (1971). Osservazioni sulla biologia dei Ceratopogonidi (*Leptoconops* spp.) nel Grossetano. Parassitologia.

[bib41] Majori G., Bettini S., Finizio E., Pierdominici G. (1970). Ricerche sui Ceratopogonidi nel Grossetano: nota IV: identificazione dei focolai di *Leptoconops* (*Holoconops*) *kerteszi* Kieffer, 1908. Riv. Parassitol..

[bib42] Messaddeq N., Fabre M., Kremer M. (1989). Étude au microscope électronique à balayage des organes sensoriels de *Culicoides nubeculosus* (Diptère: Cératopogonidé). Ann. Parasitol. Hum. Comp..

[bib43] Messaddeq N., Fabre M., Kremer M. (1990). Mise en evidence des chimiorecepteurs de lʼantenne, des palpes maxillarires et des pattes prothoraciciques chez *Culicoides nubeculosus* (Diptera: Ceratopogonidae). Bull. Soc. Fr. Parasitol..

[bib44] Mullen G.R., Mullen G., Durden L. (2002). Medical and Veterinary Entomology.

[bib45] Mullen G.R., Murphree C.S., Mullen G.R., Durden L.A. (2019). Medical and Veterinary Entomology.

[bib46] Mullens B.A., Barrows C., Borkent A. (1997). Lizard feeding by *Leptoconops* (*Brachyconops*) *califomiensis* (Diptera: Ceratopogonidae) on desert sand dunes. J. Med. Entomol..

[bib47] Perich M.J., Strickman D., Wirtz R.A., Stockwell S.A., Glick J.I., Burge R. (1995). Field evaluation of four repellents against *Leptoconops americanus* (Diptera: Ceratopogonidae) biting midges. J. Med. Entomol..

[bib48] Poinar G. (2008). *Leptoconops nosopheris* sp. n. (Diptera: Ceratopogonidae) and *Paleotrypanosoma burmanicus* gen. n., sp. n. (Kinetoplastida: trypanosomatidae), a biting midge-trypanosome vector association from the Early Cretaceous. Mem. Inst. Oswaldo Cruz.

[bib49] Polidori C., Wurdack M. (2019). Mg-enriched ovipositors as a possible adaptation to hard-skinned fruit oviposition in *Drosophila suzukii* and *D. subpulchrella*. Arthropod-Plant Interact..

[bib50] Polidori C., García A.J., Nieves-Aldrey J.L. (2013). Breaking up the wall: Metal-enrichment in ovipositors, but not in mandibles, co-varies with substrate hardness in gall-wasps and their associates. PLoS One.

[bib51] Polidori C., Jorge A., Keller A., Ornosa C., Tormos J., Asís J.D., Nieves-Aldrey J.L. (2020). Strong phylogenetic constraint on transition metal incorporation in the mandibles of the hyper-diverse Hymenoptera (Insecta). Org. Divers. Evol..

[bib52] Quicke D., Palmer-Wilson J., Burrough A., Broad G. (2004). Discovery of calcium enrichment in cutting teeth of parasitic wasp ovipositors (Hymenoptera: Ichneumonoidea). Afr. Entomol..

[bib53] Rasmussen L.D., Kristensen B., Kirkeby C., Rasmussen T.B., Belsham G.J., Bødker R., Bøtner A. (2012). Culicoids as vectors of Schmallenberg virus. Emerg. Infect. Dis..

[bib54] Raspi A., Canovai R., Loni A., Santini L. (2007). *Leptoconops* (*Holoconops*) *kerteszi* Kieffer (Diptera: Ceratopogonidae) in the coastal area of Grosseto: Eco-ethological aspects. Bull. Insectol..

[bib55] Roer R., Dillaman R. (1984). The structure and calcification of the crustacean cuticle. Am. Zool..

[bib56] Ronderos M.M., Spinelli G.M., Lager I., Díaz F. (2003). La importancia sanitaria de los jejenes del género *Culicoides* (Diptera: Nematocera) en la Argentina. Entomol. Vectores.

[bib57] Rowley W.A., Cornford M. (1972). Scanning electron microscopy of the pit of the maxillary palp of selected species of *Culicoides*. Can. J. Zool..

[bib58] Steward C.C., Atwood C.E. (1963). The sensory organs of the mosquito antenna. Can. J. Zool..

[bib59] Strickman D., Wirtz R., Lawyer P., Glick J., Stockwell S., Perich M. (1995). Meteorological effects on the biting activity of *Leptoconops americanus* (Diptera: Ceratopogonidae). J. Am. Mosq. Control Assoc..

[bib60] Sutcliffe J.F., Deepan P.D. (1988). Anatomy and function of the mouthparts of the biting midge, *Culicoides sanguisuga* (Diptera: Ceratopogonidae). J. Morphol..

[bib61] Szadziewski R., Borkent A., de Jong H. (2004). Fauna Europaea: Diptera: Nematocera, Version 1.0–27.

[bib62] Szadziewski R., Poinar G.O. (2005). Additional biting midges (Diptera: Ceratopogonidae) from Burmese amber. Pol. J. Entomol..

[bib63] Tavaré S. (1986). Some probabilistic and statistical problems in the analysis of DNA sequences. Lect. Math. Life Sci..

[bib64] Thorez A., Compère P., Goffinet G. (1992). Ultrastructure and mineral composition of the tergite cuticle of the iulid millipede *Ophyiulus pilosus* (Myriapoda, Diplopoda). Ber. Naturwiss. Med. Ver. Innsb..

[bib65] Whitsel R.H., Lauret T.H., Vickery C.A., Munsterman H.E. (1961). Proceedings of 29th Conference of the California Mosquito and Vector Control Association.

[bib66] Whitsel R.H., Schoeppner R.F. (1966). Summary of a study of the biology and control of the valley black gnat, *Leptoconops torrens* Townsend (Diptera: Ceratopogonidae). Calif. Vector Views.

